# Health-related quality of life in urban surgical emergency department patients: Comparison with a representative German population sample

**DOI:** 10.1186/1477-7525-3-77

**Published:** 2005-12-01

**Authors:** Bruno Neuner, Peter M Miller, Bodo Felsmann, Edith Weiss-Gerlach, Tim Neumann, Klaus Dieter Wernecke, Claudia Spies

**Affiliations:** 1 Dept. of Anaesthesiology, Charité-Universitätsmedizin Berlin, Campus Charité-Mitte, Berlin, Germany; 2 Center for Drug and Alcohol Programs, Medical University of South Carolina, Charleston, SC 29425, USA; 3 Institute of Medical Informatics, Charité-Universitätsmedizin Berlin, Campus Charité-Mitte, Berlin, Germany

## Abstract

**Background:**

Patients in emergency departments show a high prevalence of substance use. Quality of life is associated with substance use as well as socioeconomic status. Little is known about quality of life in substance-abusing young patients with minor trauma.

**Methods:**

An investigation in an Emergency Department in an inner city university hospital was conducted during 8 months. Overall, 1,596 patients completed the SF-36 and an established SES-questionnaire and were screened for substance use (harmful alcohol consumption (≥ 8 points in men and ≥ 5 points in women on the Alcohol Use Disorders Identification Test (AUDIT), smoking and illicit drug use). Results were compared with a representative German population sample (German Federal Health Survey 1998).

**Results:**

Median age of participants was 32 years and 61.8% were male. Mean physical component summary score (PCS) of the Short Form-36 Health Survey (SF-36) was 53.4 ± 8.3 points and significantly higher than the age and gender-stratified German Federal Health Survey-data. Mean mental component summary score (MCS) was 47.9 ± 10.0 points and significantly lower than the age and gender-stratified German Federal Health Survey-data. In Emergency Department patients, prevalence of substance use was high and harmful alcohol consumption and illicit drug use were strongly associated with impaired mental health. Education and occupational status were strongly positively associated with physical health.

**Conclusion:**

We conclude that there is a high prevalence of substance use in young patients with minor trauma and mental quality of life is impaired. Screening and brief intervention strategies to reduce substance-use associated disorders should consider these findings.

## Background

Recent investigators have shown in different settings that substance use is associated with impaired Health related Quality of Life (HRQoL). In alcohol dependent patients, Daeppen et al. [1] (1998; 147 patients in Switzerland), Mc Kenna et al. [2] (1996; 586 inpatients in Great Britain) and Volk et al. [3] (1997; 1333 primary care patients in USA) found impaired HRQoL in comparison to patients with lower severity of alcohol dependence, a normal population, and patients without alcohol disorders, respectively. In addition, HRQoL can be improved by therapeutic intervention in patients with alcohol use disorders. Kraemer et al. [4] (2002; 213 outpatient drinkers in USA) found, in their 12 months-follow-up study, that a decrease of 30%+ drinks per month led to a significant improvement in the mental component summary score of the SF-36 (p = 0.037), whereas the physical component summary score of the SF-36 showed a non-significant trend (p = 0.058).

In patients with illicit drug use, Falck et al. [5] (2000; 443 not-in-treatment crack-cocaine smokers in USA) found a negative association between drug use and all SF-36 domains except "physical functioning". No association was found between SF-36 domains and alcohol in these patients. Reid et al. [6] (2000; less than 50% cannabis users out of 1581 14–19 years-old in Australia) reported significantly lower "general health" and "vitality" in the SF-36 scores of illicit drug users compared to non-users. In drug users, too, a decrease in substance use led to improvement in HRQoL: Richter et al. [7] (2004; 100 multiple substance users in Germany) found a significant improvement in the SF-36 domains of "general health" and "physical functioning" in 48 patients at follow-up.

In smokers, Wilson et al. [8] (1999; 3010 persons older than 15 years in a population based survey in South-Australia) found, in all (light/moderate/heavy) smokers, significantly lower SF-36 domains in comparison to non-smokers, even after multiple adjustments for age, gender, socioeconomic status and number of alcoholic drinks per week. However, these investigators did not adjust for additional drug use. In a disabled population, Arday et al. [9] (2003; more than 134,000 elderly and more than 8,600 disabled in the Medical Health Outcomes Survey in USA) found significantly lower physical (PCS) respectively mental component summary score (MCS) in everyday and someday smokers compared to non-smokers. In the elderly, current smokers as well as recent quitters showed significantly lower PCS and MCS scores than non-smokers. For the disabled and elderly, MCS scores of long-term quitters were similar to those of non-smokers.

Substance use not only impairs HRQoL but increases health care utilization, i.e. emergency department (ED) visits [10,11]. Furthermore, frequent ED users in various settings were found to be characterized by improved HRQoL. Freitag et al. [12] (2005; 785 patients with chronic daily headache in 2 clinical trials in USA) showed that ED users were characterized by significantly lower SF-36 scores than non-ED users. Mandelberg et al. [13] (2000; database of more than 340,000 ED visits in the San Francisco General Hospital, USA) found frequent ED users (equal or more than 5 visits within 12 months) were more likely than non-frequent users to be homeless, poor, and diagnosed with alcohol use disorders and/or chronic illnesses. Although the authors reported no findings on HRQoL, all of these reported conditions associated with frequent ED-use are associated with impaired HRQoL.

EDs play an important role in alcohol/drug abuse screening and brief intervention (SBI) since young patients in these settings are often at an early stage of their substance abuse problems. To the best of our knowledge, little is known about the HRQoL in young patients with minor trauma in an ED setting. Therefore, the aim of this study was to evaluate the association of HRQoL, substance use (smoking, hazardous alcohol consumption and drug consumption) and socioeconomic status in patients with minor injuries in an inner-city hospital ED.

## Methods

After Ethical Committee approval and written informed consent from all participants, between December 2001 and July 2002, all consecutive patients in the surgical ED in a university-based hospital in Berlin, Germany, were included in this investigation. Survey data presented in this paper were obtained as part of a prospective intervention study to test the effects of a tailored written advice for hazardous alcohol consumption. Inclusion criteria were acute trauma and age over 18 years. We excluded patients who were either unable to give informed consent, admitted by the police, homeless, or those with insufficient knowledge of the German language. Patients were screened for substance use ("hazardous alcohol consumption" defined as ≥ 8 points (in men) and ≥ 5 points [14] (in women) on the Alcohol Use Disorders Identification Test (AUDIT) [15], "smoking" defined as all current smokers, and "use of illicit drugs" defined as the use of illicit drugs a minimum of 1 to 3 or more times within the last 12 months. Possible categories for illicit drug use were "Marihuana; Cocaine; Ecstasy; Heroin; and Other".

Socioeconomic parameters [16] were divided into binary variables including the following: (1) School education: 12 or 13-years-school education ("A-level") vs. a school education of 11 or less than 11 years ("no A-level"), (2) Family income: "equal or less than 24,000 Euro net per year" and "more than 24,000 Euro net per year", (3) Partnership: "Yes" and "No", independently of marital status, (4) Size of household: "one-person-household" and "household with more than one person", and (5) "Working" was defined as any legally paid work, either part- or full-time, including civil servant, self-employed or paid worker in a family business. "Not working" included all students, trainees, unemployed people, homemakers, and according to German law, patients in civil services or military service (except professional soldiers). "Not working" also included, retired patients, and patients engaged in non-profit voluntary work.

Surgical and trauma data such as trauma diagnosis and Injury Severity Scores (ISS) were collected after surgical treatment [17].

HRQoL was measured using the German version of the Short Form-36 Health Survey (SF-36) [18,19]. Component summary scores were calculated using the official German algorithm, using American weights to allow international comparison of results [20]. The mental component summary score (MCS) includes mental health (nervousness/depression vs. happiness and calmness), role emotional (work performance as related to emotional functioning), social functioning (performance of social activities), and vitality (energy level). The physical component summary score (PCS) includes physical functioning (performance of physical activities), role physical (work performance as related to physical functioning), pain (pain severity), and general health (overall personal health). The SF-36 questionnaire is a well accepted, generic instrument, that can be used independently of health conditions and that is applicable to patients with only minor medical disorders [19]. The SF-36-questionnaire used in this investigation is considered to be a "validated global quality of life measure" in patients with (multiple) injuries [21]. In previous investigations, the SF-36 served as an outcome measure to capture – apart from medical outcomes – patients' subjective views of their perceived quality of health.

PCS and MCS-data from this investigation were compared with data from the German Federal Health Survey 1998 [22]: The German Federal Health Survey (BGS98) began in October 1987 and included approximately 7,200 participants. It was financed by the German Ministry of Health and conducted by the Robert Koch Institute (RKI) . The RKI is a governmental institution mainly responsible for the Federal Health Reporting Service (Gesundheitsberichterstattung des Bundes, GBE), regarding surveillance, health status and health behavior of the population. BGS98 data were obtained using country-wide sampling based on randomly selected registration offices, with participants undergoing a medical check-up and an interview regarding health-relevant issues [22]. Therefore, the BGS98 is regarded as a valid representative survey, which has been used extensively as a standard measure of the heath status of the German population. However, for this investigation, original German Health Survey-1998 data (available in Public Use files) were compared with data from our study. Out of 7,124 datasets in the German Health Survey- 1998, 228 (4.0%) MCS and PCS datasets were missing; therefore analysis was based on 6,836 consecutive patients.

### Statistical analysis

All binary and categorical variables are shown as frequencies. Metric variables are shown as medians (range) when not normally distributed and median ± standard deviation when normally distributed. Statistical analysis for differences between two independent groups was performed using a Χ^2^-test for binary variables, and by using the Mann-Whitney-U-Test for not normally distributed metric variables and t-test for normally distributed metric variables. The correlative structure between MCS respectively PCS as dependent variables, and covariates (age, gender, ISS trauma-score, socioeconomic status (high school degree, family income, partnership, size of household, employment status), and substance use (harmful alcohol consumption, smoking and illicit drug use)) was analyzed using linear multiple regression with stepwise variable selection procedure. An α-level of 0.05 was used as the level of significance. To compare PCS and MCS between emergency department patients and data in the German Federal Health Survey, a multifactor analysis of variance (ANOVA) was conducted with "setting", "gender" and "age-group" as fixed factors. To test for homogeneity of variances, the Levene's Test for Equality of Variances was administered, in order to adapt the α-level in case of heterogeneity. Statistical analyses were performed using SPSS (Microsoft SPSS for Windows, version 12.0).

## Results

Between December 2001 and July 2002, 1,779 consecutive ED patients were included in the study. Of these, 39 SF-36-questionnaires were incomplete. Of the remaining 1,740 datasets, 144 were incomplete due to missing in the socioeconomic parameters. Therefore, the final database consisted of 1,596 patients (89.7% of 1,779).

Of these 1,596 consecutive patients, 61.8% were men and the overall median age was 32 (18 – 89) years. More than 80% of all patients were characterized by minor trauma (ISS = 1). The maximum ISS was 10 points, suggesting that this population was a homogenous group with minor trauma. In regard to specific ICD-10 diagnosis, 14.1%, respectively 4.2% of all patients had trauma diagnoses belonging to the S0-and S1-Group (Head respectively neck injuries, mainly head lacerations), another 4.1% respectively 1.3% showed diagnosis of the S2, respectively S3-group, which meant injuries of the thorax, respectively abdomen, overall 73.5% of diagnoses belonged to the S4 – S9 group, with 35.4% diagnoses of the upper extremities and the shoulder and 38.1% injuries of the lower extremities and the hip. Most of these injuries were bruises and lacerations, fractures of finger, hand or forearm ore ankle torsions. A total of 43 patients (2.7%) showed multiple injuries. Detailed socioeconomic and substance abuse parameters are presented in Table 1. Men in comparison to women were less likely to have an A-level and were more often employed and living alone. The prevalence of hazardous alcohol consumption, smoking and illicit drug use was significantly higher in men than in women. Concerning HRQoL, overall physical component summary score (PCS) was 53.4 ± 8.3 points with higher PCS in men (53.9 ± 7.5 points) than in women (52.5 ± 9.3 points), p = 0.003). The mental component summary score (MCS) was 47.9 ± 10.0 points with higher MCS in men (48.5 ± 9.4 points) than in women (46.9 ± 10.8 points), p = 0.003).

**Table 1:** Basic characteristics, socio-economic status, substance use and Health Related Quality of Life in all patients

Variable	all n = 1596	male n = 986 61.8%	female n = 610 38.2 %	p
Age (1) (years)	32 (18 – 89)	32 (18 – 78)	31 (18 – 89)	0.306
ISS (%) >1/1 point	18.3/81.7	19.7/80.3	16.1/83.9	0.070
**Socioeconomic status**				
A-Level (%) no/yes	43.9/56.1	46.2/53.8	40.2/59.8	0.017
Being employed(%) no/yes	36.4/63.6	33.7/66.3	40.8/59.2	0.004
Family income (%)* ≤24,000 €/>24,000 €	62.2/37.8	62.1/37.9	62.5/37.5	0.876
Partnership (%) no/yes	54.3/45.7	54.7/45.3	53.6/46.4	0.680
Size of household (%) 1/>1 person	36.8/63.2	39.7/60.3	32.3/67.7	0.003
**Substance use**				
Hazardous alcohol consumption # (%) yes/no	24.7/75.3	26.5/73.5	22.0/78.0	0.043
Illicit drug use (%) yes/no	21.7/78.3	25.6/74.4	15.6/84.4	< 0.001
Smoking (%) yes/no	45.7/54.3	51.7/48.3	36.1/63.9	< 0.001
**Health Related Quality of Life (HRQoL)**				
PCS (2)	53.4 ± 8.3	53.9 ± 7.5	52.5 ± 9.3	0.003
MCS (2)	47.9 ± 10.0	48.5 ± 9.4	46.9 ± 10.8	0.003

(1): median (range); (2): mean ± standard deviation; ISS: Injury Severity Score; A-level: "yes": 12 or 13 years of school education, "no": 11 or less years of school education; *; family income net per year; #: hazardous alcohol consumption "yes": (in men): AUDIT = 8 – 40 points, (in women): AUDIT = 5 – 40 points, "no": (in men): AUDIT = 0 – 7 points, (in women): AUDIT = 0 – 4 points; PCS: physical component summary score of the SF-36; MCS: mental component summary score of the Sf-36.

When entering the anthropometric, injury, socioeconomic and substance use parameters presented in table 1 into 2 regression models with the physical component summary score (Table 2) and the mental component summary score (Table 3) as dependent variables, we found that male gender, A-level, and being employed were positively associated with a significant increase in physical HRQoL. However, with every life year there was a small but significant decrease in physical HRQoL (+ every additional life year: -0.2 ± 0.02 points in PCS, p < 0.001). Concerning mental health, with every life year there was a small but significant increase in mental HRQoL (+ every additional life year: +0.05 ± 0.02 points in MCS, p = 0.012). Other factors positively associated with mental health were male gender, a yearly income above 24,000€, and living in a more-person-household. In this investigation, injuries with more than 1 ISS-point were associated with a higher MCS. All substance use parameters (with illicit drug use showing the strongest association (-2.74 ± 0.64 points, p < 0.001)) were negatively associated with mental health, although smoking showed a non-significant trend (-0.87 ± 0.52 points, p = 0.097).

**Table 2:** Results of the linear multiple regression model (with stepwise variable selection procedure), dependent variable = physical component summary score of the SF-36 (PCS)

**Covariates**	**β_x_-coefficient**	**se (β_x_)**	**p**
β_*0*_	57.03	1.02	< 0.001
Age (+ 1 year) (β_*1*_)	-0.20	0.02	< 0.001
Male gender (β_*2*_)	0.97	0.40	0.015
A-level (β_*3*_)	2.88	0.39	< 0.001
Being employed (β_*4*_)	2.73	0.40	< 0.001
Household > 1 person (β_*7*_)	-0.72	0.39	0.071

Model = β_0 _+ β_1 _* age + β_2 _* male gender + β_3 _* A-level + β_4 _* being employed + β_5 _* income > 24,000€/year + β_6 _*partnership + β_7 _* household > 1 person + β_8 _* hazardous alcohol consumption + β_9 _* illicit drug use + β_10 _* smoking + β_11 _* ISS > 1 point.

A-Level: 12 or 13 years of school education, ISS = Injury Severity Score.

**Table 3:** Results of the linear multiple regression model (with stepwise variable selection procedure), dependent variable = mental component summary score of the SF-36 (MCS)

**Covariates**	**β_x_-coefficient**	**se (β_x_)**	**p**
β_*0*_	39.28	2.02	< 0.001
Age (+ 1 year) (β_*1*_)	0.05	0.02	0.012
Male gender (β_*2*_)	2.22	0.51	< 0.001
Income > 24,000 €/year (β_*3*_)	1.52	0.53	0.004
Household > 1 person (β_*7*_)	1.31	0.51	0.010
Hazardous alcohol consumption (β_*8*_)	-1.84	0.59	0.002
Illicit drug use (β_*9*_)	-2.74	0.64	< 0.001
Smoking (β_*10*_)	-0.87	0.52	0.097
ISS > 1 point (β_*11*_)	1.251	0.63	0.048

Model = β_0 _+ β_1 _* age + β_2 _* male gender + β_3 _* A-level + β_4 _* being employed + β_5 _* income > 24,000€/year + β_6 _*partnership + β_7 _* household > 1 person + β_8 _* hazardous alcohol consumption + β_9 _* illicit drug use + β_10 _* smoking + β_11 _* ISS > 1 point.

A-Level: 12 or 13 years of school education, ISS = Injury Severity Score

**Figure 1:**
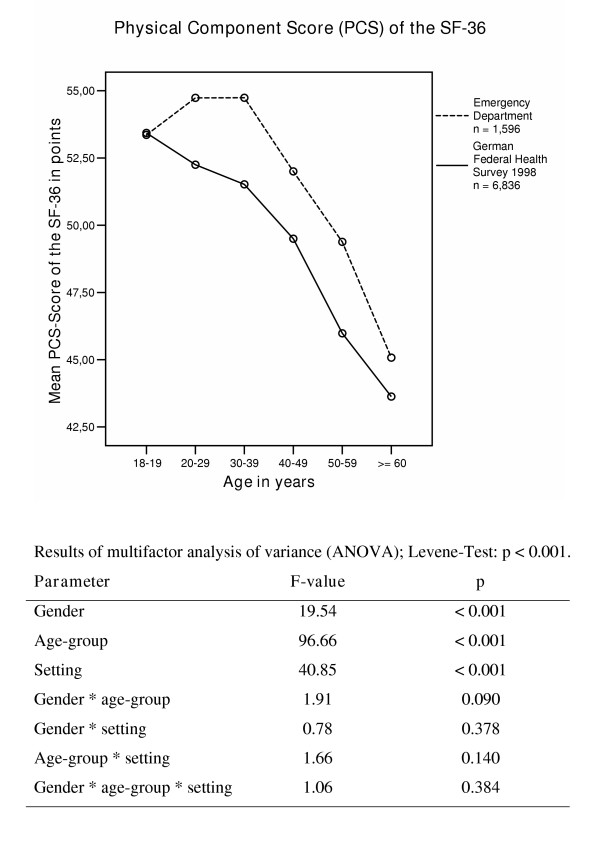
Physical Component Summary Score (PCS) in different age-groups in Emergency Department patients and in participants in the German Federal health survey 1998.

The physical HRQoL in ED patients in comparison to a representative sample in Germany (n = 6,836) is shown in Figure 1. ED patients, in all age groups except 18–19-year-olds, showed a better physical HRQoL than the representative German population sample. Taking both samples together, age explained the most variance in the overall data, followed by "setting". Gender differences explained less variance in the model.

**Figure 2:**
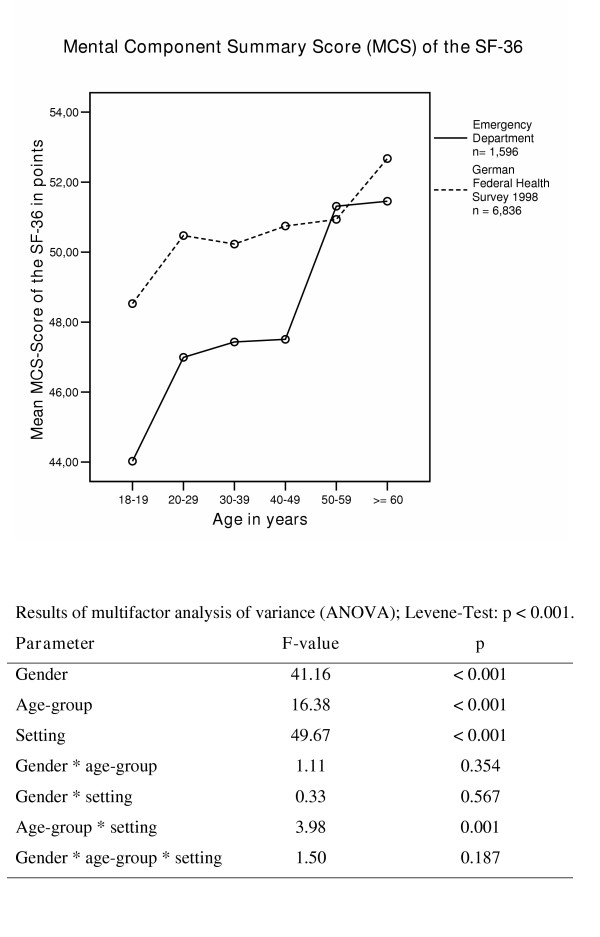
Mental Component Summary Score (MCS) in different age-groups in Emergency Department patients and in participants in the German Federal health survey 1998.

The mental HRQoL in ED patients in comparison to a representative sample in Germany (n = 6836) is shown in Figure 2. MCS scores in ED patients in all but one age-group (50 – 59 years) were found to be lower compared to the representative German sample. Concerning mental health, "setting" was the variable explaining the most variance when considering both populations together. Gender differences explained the second highest proportion of variance, followed by age.

## Discussion

The most important result of this study was the lowered mental quality of life in young ED patients with minor trauma in comparison to a representative German population sample. Physical quality of life in ED patients was even better than in the representative German population sample. In ED patients the absence of substance use parameters such as illicit drug use and harmful alcohol consumption as well as male gender, older age and a high family income were positively associated with mental health. Better physical health in ED patients was positively associated - apart from male gender and younger age – with protective socioeconomic parameters such as A-level and being employed.

Mental HRQoL in young ED patients with minor trauma was strongly negatively associated with substance use parameters, especially alcohol and illicit drug use. These results reflect previous findings in different settings [1-7]. Smoking alone showed only a negative tendency. Our findings suggest as well that family income and size of household were the independent relevant factors associated with mental HRQoL. A higher income may provide more opportunities to meet personal demands and may lead to higher satisfaction and thus improved mental HRQoL. Male ED patients showed better mental HRQoL than female ED patients; better mental HRQoL in men in comparison to women was reported in BGS-98-data in Germany [23] as well as in data from the Whitehall II Study [24].

Concerning physical health, in this predominant young population (median age = 32 years), substance use parameters were not associated with physical health. One explanation might be that these patients were too young to be, for example, seriously affected by alcohol-related disorders which appear in later years. In an other investigation we found a median age of 42 years in severely injured alcohol dependent patients [25], a median age of 47 years was reported in 167 patients with alcohol associated liver cirrhosis [26] and a median age of patients with alcohol associated tumors of the upper digestive tract who underwent tumor resection was found to be 56 years [27]. Therefore in these young trauma patients, substance use parameters may not (yet) be important factors in affecting physical HRQoL.

However, socioeconomic data were associated with physical HRQoL – apart from gender and age, which are both established predictors of physical HRQoL (gender: in both, BGS98 [23] and Whitehall II study [24], with increasing age an impaired physical HRQoL was found. In the USA, Arday et al. [9] (2003, more than 130,000 elderly respectively more than 8,600 impaired) found a decrease in physical HRQoL of 0.45 points respectively 0.29 points for every additional life year. Not surprisingly, in our investigation, a decrease of 0.2 ± 0.02 points in physical HRQoL was found with every additional life year. In ED patients, a higher educational level and being employed was associated with improved physical HRQoL. Hemingway et al. [24] (1997, more than 10,000 participants in the Whitehall II study in GB) found that lower employment grade was positively associated with lower physical HRQoL. In both gender, all SF-36 domains in the BGS98-data [23] (Kurth & Ellert 2002, German representative population sample of 7,200 participants) – even after multiple adjustments for age, region and community size – were positively associated with social class. In our investigation in young trauma patients, no association was found between physical HRQol and income. Woolf et al. [28] (1998, 555 patients in an inner-city family practice center in USA) found in patients with a yearly income of less than $15,000 lower physical function scores then those repeated nationally for patients with hypertension, diabetes, depression or recent myocardial infarction. However, these patients were older then the trauma population in this investigation.

The lower mental HRQoL in younger ED patients in comparison with the representative BGS-data may be partly due to the high prevalence of substance use parameters in ED patients. Data are not easily comparable since the AUDIT-questionnaire was not used in the BGS-Survey and illicit drug use was evaluated using a different algorithm. However, evidence from other representative population surveys in Germany suggest lower population-based prevalence rates (alcohol: dependence 3%; misuse 5% and overall at risk consumption 16% in 2000; smoking: 27% smoking prevalence 2003 in the population equal or older than 15 years; illicit drug use: 12-month-prevalence: 6.5% of the population in West-Germany and 5.2% in East-Germany in 2000) [29].

To the best of our knowledge, no comparable data are available concerning our findings on better physical HRQoL. There are several investigations on trauma patients in urban settings. Sims et al. [10] (1989, 501 survivors of violent trauma in USA) used the terminus "urban trauma" to characterize young, male patients with high prevalence of substance use and unemployment, who showed 44% trauma recidivism and a 5-years-mortalityof 20% at follow-up. Other investigators in urban (Smith RS et al. [30], 1992: 342 trauma recidivists in an urban trauma center, USA; Reiner DS et al. [31], 1990: 150 consecutive admissions in a level 1-Trauma-Center, USA) as well in rural settings (Poole GV et al. [11], 1993: 200 trauma patients in a  university hospital and level I trauma center, USA; Sayfan and Berlin [32], 1997: 100 trauma cases in northern Israel) reported young age, male gender, previous admission for trauma and positive alcohol blood level during admission as being risk factors for recurrent trauma.

None of these investigations reported data on HRQoL. However, even this population with minor trauma was characterized by parameters (male gender, young age, high prevalence of substance use) which were found to be associated with more severe trauma and even trauma recidivism. Assuming that in our investigation we received information on patients at a very early stage of a worsening career of substance use and repetitive injuries, we consider the younger of these patients being "sensation seekers" [33] with as yet unaffected physical health but impaired mental health. At this early stage, patients may consider themselves "invincible" and their substance use as well as their injuries may be the result of unaffected physical health in combination with impaired mental health and impaired coping strategies. We found substance use, especially alcohol consumption, in these ED patients associated with poor coping capability [34]. Thus, minor injuries in our study population would not be the result of random effects, but triggered by an impaired mental HRQoL as well as impaired coping ability in combination with unaffected physical health that may lead to risky behaviors such as substance use.

## Conclusion

In comparison to the general population young patients treated for minor trauma in an urban ED showed an impaired mental health related quality of life in combination with an improved physical health related quality of life. Together with a high prevalence of substance use, this cluster may play a causal role in unintentional injuries. Screening and brief intervention programs on substance use in Emergency Departments should consider this finding and appropriate health promoting strategies focusing on improvement of mental health should be integrated in SBI.

## Authors' contributions

Neuner B contributed in the design of the study, coordinated data collection, training of the study staff, made the data analysis and wrote the article, Miller P helped to design the study and revised the article critically for important intellectual content, Bodo Felsmann made substantial contributions to data collection and revised the article critically, Edith Weiss-Gerlach and Tim Neumann contributed in the design of the study, coordinated data collection, training of the study staff and revised the article critically, Klaus Dieter Wernecke contributed substantially in statistical analysis and interpretation and Claudia Spies designed and coordinated the study, revised the article critically and gave final approval of the version to be published.
